# The mythological and archaeological perspectives on penectomy and orchiectomy: The case of Cybele and Attis

**DOI:** 10.1111/andr.70040

**Published:** 2025-04-07

**Authors:** Coskun Kaya

**Affiliations:** ^1^ Department of Urology Health Science University Eskisehir City HARH Eskişehir Türkiye

**Keywords:** Attis myth, castration, cybele cult, mythology, orchiectomy, penectomy

## Abstract

Castration has been a significant theme in mythology, religious traditions, and historical practices, often symbolizing transformation, sacrifice, and divine punishment. While the term is frequently associated with orchiectomy (removal of the testes), this study argues that penectomy (removal of the penis) must also be considered, particularly in myths where the symbolic weight of castration extends beyond mere fertility loss. The myth of Cybele and Attis serves as a compelling example, raising questions about the intended nature of Attis' self‐mutilation and its implications.

## 1 INTRODUCTION

*Castration*, in its strict medical and biological definition, refers specifically to the removal or inactivation of the gonads (testes in males, ovaries in females). However, historical and mythological texts frequently use the term “castration” more broadly and sometimes inaccurately, encompassing various forms of genital mutilation. To clarify this distinction, genital mutilation can be categorized into three distinct forms:

1. **Orchiectomy**—Removal of the testes.
2. **Penectomy**—Removal of the penis.
3. **Complete genital removal**—Removal of both the penis and testes.

Castration has been practiced throughout history for various cultural, religious, and social reasons. In ancient societies, castration was often associated with servitude, religious devotion, or political power. Eunuchs, men who had undergone castration, frequently served as court officials, harem guards, or religious functionaries in civilizations such as Mesopotamia, China, and Byzantium.^1^ In many cases, these individuals were entrusted with significant responsibilities because they were perceived as loyal, non‐threatening, and incapable of producing heirs who might challenge ruling authorities.

In a religious context, castration was sometimes seen as an act of piety and spiritual transformation. Various myths and religious traditions include narratives of divine or ritualistic castration, reflecting the broader symbolism of self‐sacrifice and rebirth. For instance, in Greek ***mythology***, the Titan ***Cronus*** castrated his father ***Uranus*** with a sickle, separating the sky from the Earth and allowing the cosmos to be structured. The severed genitals of Uranus were cast into the sea, giving birth to ***Aphrodite***, the goddess of love and beauty. This act symbolized both destruction and creation, showing how castration could lead to transformation and the emergence of new divine orders.^2^ Similarly, in ***Hittite mythology***, the god ***Kumarbi*** bit off the genitals of the sky god ***Anu***, which led to the birth of various deities, including the storm god Teshub.^3^ This narrative, much like that of Uranus and Cronus, demonstrates how castration myths often signify the transfer of power and generational shifts among deities. In ***Hindu mythology***, the deity ***Shiva*** is sometimes associated with the concept of asceticism and sexual renunciation. While Shiva himself is not castrated, certain sects of devotees, such as the ***Hijras***, practice ritual castration as a means of devotion and spiritual transformation, aligning themselves with divine androgyny.^4^ Similarly, in ancient Egyptian mythology, the god Osiris was said to have been dismembered by his brother Set, including the loss of his genitals, which were later reconstructed by Isis to conceive Horus. This myth reflects the intersection of castration, death, and rebirth within Egyptian religious thought.^5^ Christian tradition also contains references to castration. Origenes, one of the early Christian theologians, is believed to have castrated himself in an effort to resist sexual temptation and achieve spiritual purity.^6^ In addition, the story of Abelard, a medieval philosopher and theologian, highlights the link between castration, punishment, and the renunciation of sexual desire. Abelard was forcibly castrated after a romantic relationship with his student Heloise was discovered, symbolizing the ultimate price of forbidden love and reinforcing the moral codes of his time.^7^ These myths demonstrate how castration was not only a physical act but also a powerful symbolic and religious gesture across various cultures, often linked to themes of ***power***, ***sacrifice***, ***creation***, and divine succession.

In mythological contexts, distinguishing between these forms of genital mutilation is crucial as each carries significantly different symbolic meanings and physiological consequences. Historically, many ancient sources have used the term “castration” loosely, without clearly differentiating among these specific procedures. Consequently, scholarly analysis of myths involving genital mutilation should carefully consider which particular form is being referenced or symbolically implied, rather than defaulting to the assumption that castration always entails orchiectomy. Understanding these distinctions is critical for analyzing ancient myths and religious practices involving castration. Most historical and religious sources refer to castration in a broad sense, often without specifying the exact form. However, the psychological and physiological consequences of orchiectomy, penectomy, and complete genital removal differ significantly, shaping the symbolic meaning attributed to castration in various cultural contexts. In archaeological and mythological studies, castration is generally assumed to mean orchiectomy. However, this reductionist view overlooks the possibility that penectomy may have been equally relevant in certain religious and ritualistic contexts. While most historical and religious texts imply orchiectomy when describing castration, it is crucial to consider that some cases may have involved penectomy instead. One of the most intriguing examples of religiously motivated castration is found in early Christianity. Some of the earliest Christian teachings included the practice of castration, not only as a symbolic act of devotion but as a literal gesture of spiritual purity. The Gospel of Matthew mentions that some individuals were “made eunuchs by men,” referring to forced castration, while others “made themselves eunuchs for the sake of the kingdom of heaven,” implying voluntary castration as a form of religious commitment and asceticism.^8^ Although this passage has historically been interpreted as referring to castration, the exact nature of genital mutilation—whether orchiectomy, penectomy, or complete genital removal—is not explicitly detailed, highlighting ambiguity in the historical and religious interpretation.^9^ Interestingly, even circumcision was at times equated with castration and punished accordingly. The Roman Emperor Hadrian was the first to equate circumcision with castration, not for religious reasons but because of the superficial similarity of the procedures. This policy contributed to a serious Jewish insurrection at the time. Circumcision was thus viewed as a form of castration and punished equally under Roman law. This demonstrates how the definition and perception of castration extended beyond the removal of reproductive function to include any form of genital modification.^10^ Although historical narratives generally focus on orchiectomy as the primary form of castration, penectomy or complete genital removal could also have occurred, depending on the symbolic intent and context of the myth. Perhaps the clearest example of this distinction is found in the myth of Attis and Cybele.^11^ While there are different versions of the Attis myth, the most consistent and widely accepted narrative portrays Attis as being driven into madness by Cybele's divine wrath, leading to his self‐castration beneath a pine tree. However, as Casadio highlighted, there are at least two major versions of the myth: the Phrygian version, where Attis emasculates himself completely and dies as a result, and the Hellenistic version, where Attis survives and becomes a eunuch‐priest devoted to Cybele.^5^

This study aims to explore how the interpretation of castration in myths should not be limited to the removal of testes alone. The possibility of penectomy, particularly in the case of Attis, where the nature of his self‐mutilation significantly impacts the myth's symbolic and cultural implications, should also be considered. For this purpose, the version presented by Ovidius has been used, which frames Attis' self‐castration as a direct consequence of Cybele's divine punishment, focusing on the symbolic and psychological implications of this act.^12^

### 1.1 The myth of Cybele and Attis^12^

**FIGURE 1:**
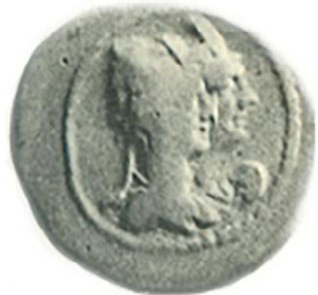
Coin depicting Cybele and Attis in profile, illustrating their divine association and religious significance. (*Source*: Vermaseren, M.J. (1987). Corpus Cultus Cybelae Attidisque (CCCA), No. 2563–64.).

This myth is believed to have taken place near the ancient city of Pessinus, which is located within the present‐day borders of Eskişehir in Türkiye. Cybele, a powerful mother goddess associated with fertility, mountains, and wild nature, was deeply revered in Phrygian and later Greco–Roman religious traditions. Attis, described as a beautiful youth, was either her consort or a mortal priest dedicated to her service. According to the myth, Cybele and Attis shared a profound and sacred love, which symbolized the union of divine power and mortal devotion. Attis' loyalty and love for Cybele were seen as the foundation of their bond, reinforcing Cybele's authority as both a maternal and romantic figure. Their divine connection was so influential that it continued to hold cultural and symbolic significance in later periods. As seen in Figure 1, coins were minted in honor of this sacred relationship, reflecting its lasting impact on religious and social traditions. However, the most common version of the myth narrates that Attis was betrothed to a mortal woman, angering Cybele, who either directly or through divine intervention drove him into madness. The betrayal of Attis not only shattered their sacred connection but also represented a rejection of Cybele's dominance and exclusive devotion.

Cybele's wrath was not merely an expression of divine jealousy but a reflection of her absolute authority over those devoted to her. As the embodiment of untamed nature and maternal power, Cybele demanded unwavering loyalty from her followers. Attis, by choosing a mortal woman, symbolically rejected his exclusive servitude to the goddess. His perceived betrayal invoked a punishment that was both immediate and irreversible.

**FIGURE 2:**
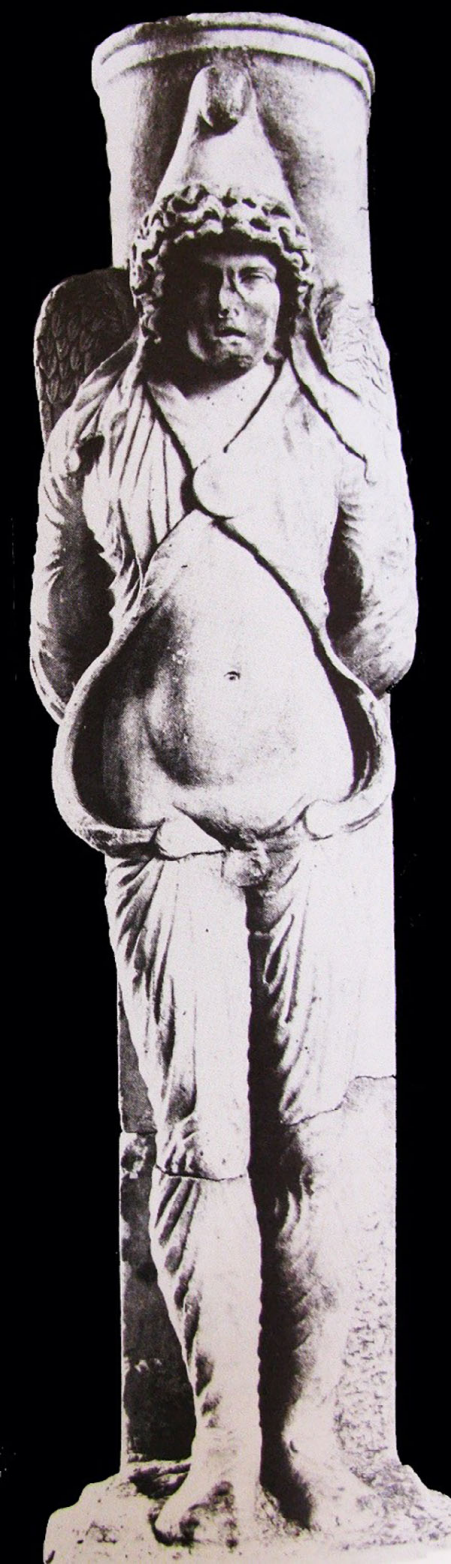
Statue of Attis from Kyzikos‐Metroon, depicted wearing a Phrygian cap and traditional attire, symbolizing his role as Cybele's servant and the ritualistic significance of his self‐castration. (*Source*: Vermaseren, M. J. (1987). *Corpus Cultus Cybelae Attidisque (CCCA)*. No. 284 / Lev. LX. Leiden: Brill.).

Cybele did not merely cast Attis aside but ensured that his punishment was absolute. As a consequence of her divine fury, Attis was driven into a state of uncontrollable madness, culminating in his act of self‐castration beneath a pine tree. This act was not just an impulsive self‐mutilation but a direct result of Cybele's divine intervention, marking his eternal separation from mortal pleasures and desires. His self‐mutilation was not only a loss of his reproductive ability but also a symbolic submission to Cybele's divine order. Through this act, he was stripped of his conventional masculinity, ensuring that he would no longer belong to the world of ordinary men. An important visual representation of Attis was the statue from Kyzikos‐Metroon (Figure 2). The figure is depicted wearing a Phrygian cap, reinforcing the connection to his Phrygian origin and his status as Cybele's servant.^13^ The prominent positioning of the hands and the physical features of the statue suggest symbolic references to both fertility and emasculation, highlighting the dual nature of Attis' myth—his initial virility and subsequent castration. This depiction aligns with the broader religious and ritualistic themes of the Cybele and Attis cult, where self‐castration was seen as an act of devotion and transformation.

Cybele, stricken with grief yet also asserting her dominance, deified Attis and ensured that he would be resurrected every spring, mirroring the cycle of vegetation and renewal. This myth became the foundation for annual religious festivals, including the Hilaria, where devotees mourned Attis' death and later celebrated his rebirth.

The myth also provided the religious justification for the self‐castration of Cybele's priests, the Galli.^14^ They underwent voluntary castration as an act of devotion and transformation, symbolizing their complete dedication to the goddess and renunciation of conventional masculinity. Their ritual mirrored Attis' fate, reinforcing Cybele's authority and the idea that submission to the divine required the severance of mortal desires.

## 2 DISCUSSION

A crucial question that arises from the myth is whether Attis' castration involved orchiectomy (removal of the testes) or penectomy (removal of the penis). If Cybele intended to punish Attis by diminishing his sexual desire, orchiectomy would have led to a significant reduction in testosterone, decreasing his attraction toward women. However, if penectomy was the method of castration, Attis would still experience sexual desire but be physically incapable of engaging in intercourse. This raises the possibility that Cybele, wanting to ensure Attis' suffering and regret, may have orchestrated a punishment that left him with unfulfilled longing, making penectomy a more fitting interpretation of his fate.

The severity of this punishment aligns with Cybele's role as an uncompromising deity demanding absolute devotion. Unlike other myths where castration is used as a means of power transfer (e.g., Cronus castrating Uranus), in the Attis myth, castration serves as a mechanism of control, submission, and suffering. The psychological impact of a punishment where desire persists but cannot be acted upon introduces an additional layer of torment, reinforcing Cybele's dominance over Attis even beyond death.

Unfortunately, due to the oral transmission of these myths before they were written down in later periods, we lack definitive textual evidence clarifying the exact nature of Attis' self‐mutilation. Although many mythological narratives describing castration often assume orchiectomy, possibly due to its known physiological effects, in the case of Cybele and Attis, a punishment designed to induce both physical suffering and emotional torment aligns more closely with penectomy than orchiectomy. This perspective suggests that the myth's underlying intent was not only about transformation but also about instilling a profound sense of remorse in Attis, ensuring that his transgression against Cybele would never be repeated.

A more recent historical parallel to the Attis myth can be found in the case of Peter Abelard.^7^ Unlike Attis, whose story was rooted in oral tradition and religious symbolism, Abelard's castration was supported by historical records and artistic depictions. However, despite the availability of more concrete evidence, the exact nature of Abelard's castration remained unclear. Recently, it has been suggested that Abelard's punishment likely involved not only orchiectomy but also penectomy, reinforcing the possibility that his castration extended beyond the simple removal of the testes.^14^

The practice of ritual castration among the Galli priests, who followed in Attis' footsteps, raises another important question: Did they undergo orchiectomy or penectomy? Historical and archaeological records suggest that self‐castration within the Cybele cult was not uniform, but the fact that the act was seen as a total renunciation of masculinity may hint that it went beyond simple orchiectomy. If the goal was to completely sever ties with conventional male identity and sexual function, penectomy may have been a more prevalent form of self‐sacrifice than previously assumed. Perhaps what has been historically described as castration may, in fact, represent early forms of circumcision, as discussed by Mordeniz et al.^9^ Their analysis suggests that circumcision might have originated as a less invasive but symbolically equivalent alternative to penectomy and castration. This hypothesis reflects how rituals of bodily modification evolved from extreme forms of genital removal to more symbolic yet equally meaningful practices.

Moreover, medical evidence suggests that removal of the testes (orchiectomy) after puberty would not necessarily eliminate sexual desire. While it would prevent procreation and inhibit secondary sexual characteristics, erection and ejaculation would still be possible due to continued function of the prostate gland and seminal vesicles. This supports the theory that penectomy, rather than orchiectomy, may have been a more fitting form of punishment in myths where the goal was not only to eliminate sexual function but to ensure that desire remained while the ability to act upon it was permanently removed.

## 3 CONCLUSION

Castration, although a frequent theme in myths and religious traditions, has often been understood primarily as orchiectomy, largely because of the lack of detailed written records in many ancient narratives. The oral transmission of myths before their documentation has contributed to this narrow perception, leading scholars to assume that castration in mythological contexts simply meant the removal of the testes. Another reason could be that archaeologists studying this topic may not fully understand the difference between penectomy and orchiectomy. However, as seen in the Cybele and Attis myth, the intended meaning of castration varies depending on the message and symbolic weight of the myth itself. In cases where the myth emphasizes loss, submission, and eternal suffering, penectomy may be the more fitting interpretation. The distinction between orchiectomy and penectomy is crucial in understanding the psychological and symbolic impact of these myths, as one removes sexual function entirely, while the other allows desire to persist without the ability to act upon it. For this reason, researchers studying myths involving castration should go beyond traditional assumptions and carefully analyze the thematic and symbolic context of each narrative. Instead of defaulting to orchiectomy, scholars must consider penectomy as a possibility, particularly when the myth's intended meaning aligns with permanent emasculation, psychological suffering, and divine punishment. By expanding the framework through which castration is examined in mythology, a more nuanced understanding of gender, power, and divine control in ancient narratives can be achieved.

## AUTHOR CONTRIBUTIONS

*Study conception and design*: Coskun Kaya. *Analysis and interpretation of results*: Coskun Kaya. *Draft manuscript preparation*: Coskun Kaya. All authors reviewed the draft manuscript and approved the final version of the paper.

## CONFLICT OF INTEREST STATEMENT

The author declares no conflicts of interest.
